# Swiss students and young physicians want a flexible goal-oriented GP training curriculum

**DOI:** 10.1080/02813432.2018.1487582

**Published:** 2018-06-26

**Authors:** Zsofia Rozsnyai, Kali Tal, Marius Bachofner, Hubert Maisonneuve, Cora Moser-Bucher, Yolanda Mueller, Nathalie Scherz, Sebastien Martin, Sven Streit

**Affiliations:** a Institute of Primary Health Care Bern (BIHAM), University of Bern, Bern, Switzerland;; b SempacherseePraxis, Oberkirch, Switzerland;; c Primary Care Unit, Faculty of medicine, University of Geneva, Geneva, Switzerland;; d Center for Primary Care Medicine, University of Basel, Basel, Switzerland;; e Department of Ambulatory Care and Community Medicine, University Institute of Family Medicine, University of Lausanne, Lausanne, Switzerland;; f Institute of Primary Care, University of Zurich, University Hospital Zurich, Zurich, Switzerland;; g Arud Centres for Addiction Medicine, Zurich, Switzerland;; h Department of Ambulatory Care and Community Medicine, French-speaking Switzerland Curriculum of Family Medicine, University of Lausanne, Lausanne, Switzerland

**Keywords:** GP training, postgraduate training curriculum in general practice: GP shortage, vocational training in general practice, primary care training, family medicine postgraduate training

## Abstract

**Background:** A growing shortage of general practitioners (GPs), in Switzerland and around the world, has forced countries to find new ways to attract young physicians to the specialty. In 2017, Switzerland began to fund hundreds of new study places for medical students. This wave of young physicians will soon finish University and be ready for postgraduate training. We hypothesized that an attractive postgraduate training program would encourage interested young physicians to pursue a GP career.

**Methods:** This is a cross-sectional survey of young physicians from the Swiss Young General Practitioners Association (JHaS), members of Cursus Romand de médecine de famille (CRMF), and all current medical students (5^th^ or 6^th^ years) (n = 554) in Switzerland, excluding students indicating definitely not to become GPs. We asked all if they were likely to become a GP (Likert: 1-10), and then asked them to score general features of a GP training curriculum, and likely effects of the curriculum on their career choice (Likert scale). They then rated our model curriculum (GO-GP) for attractiveness and effect (Likert Scales, open questions).

**Results:** Most participants thought they would become GPs (Likert: 8 of 10). Over 90% identified the same features as an important part of a curriculum (“yes” or “likely yes”): Our respondents thought the GO-GP curriculum was attractive (7.3 of 10). It was most attractive to those highly motivated to become GPs. After reviewing the curriculum, most respondents (58%) felt GO-GP would make them more likely to become a GP. Almost 80% of respondents thought an attractive postgraduate training program like GO-GP could motivate more young physicians to become GPs.

**Conclusions:** Overall, medical students and young physicians found similar features attractive in the general and GO-GP curriculum, regardless of region or gender, and thought an attractive curriculum would attract more young doctors to the GP specialty.

**Key points**
- An attractive postgraduate training program in general practice can attract more young physicians to become GPs.
- In this study cross-sectional survey including medical students (n = 242) and young physicians (n = 312) we presented general features for a curriculum and a model curriculum for general practice training, for evaluation of attractiveness to our study population.
- General practice training curriculum provides flexibility in choice of rotations, access to short rotations in a wide variety of medical specialties, training in specialty practices as well, mentoring and career guidance by GPs and guidance in choosing courses/certificate programs necessary for general practice.
- These findings help building attractive postgraduate training programs in general practice and fight GP shortage.

## Introduction

In Switzerland, as in many other European countries, we face a shortage of GPs [1]. In 2017, to address the problem of low numbers, Switzerland increased its capacity for educating young physicians by funding hundreds of new places for medical students. It now needs to make the GP profession more attractive to the younger generation [2–4]. One strategy has been to expose medical students to the specialty from their first year. Another approach has been to build postgraduate training programs in general practice [5,6]. The postgraduate training period is particularly influential because most young doctors decide to become a GP during that time [7]. When we looked at other developed countries, we saw that recognizing general practice as a specialty and implementing well-organized and structured postgraduate training programs in general practice were key factors in improving primary care [8]. The Netherlands, for example, has done so [9].

We hypothesized that building and implementing an attractive curriculum could attract to and retain more young physicians in general practice [10–12]. If young physicians train full-time, they can get board-certified in five years [13], but the average time to board certification in general internal medicine is about 6.5 years [14]. Today, physicians can be board certified without participating in a curriculum, but a curriculum may help young physicians stay on track and expose them earlier to the specialties needed for general practice.

We set out to define the components of such a curriculum by first surveying the literature and existing national programs to develop a questionnaire and a model curriculum (GO-GP), and then to survey medical students and postgraduates to determine the features they thought most attractive [15,16].

## Material and methods

### Design

Cross-sectional study of Swiss medical students and young physicians.

### Study population

To represent medical students, we surveyed students in their final (6^th^) year at all 5 Swiss medical universities, since late-year students were most likely to have the most clinical experience and the firmest ideas about their career. We surveyed 5^th^ year students due to an ongoing study with 6^th^ year students at one major Swiss university.

**Figure 1.**
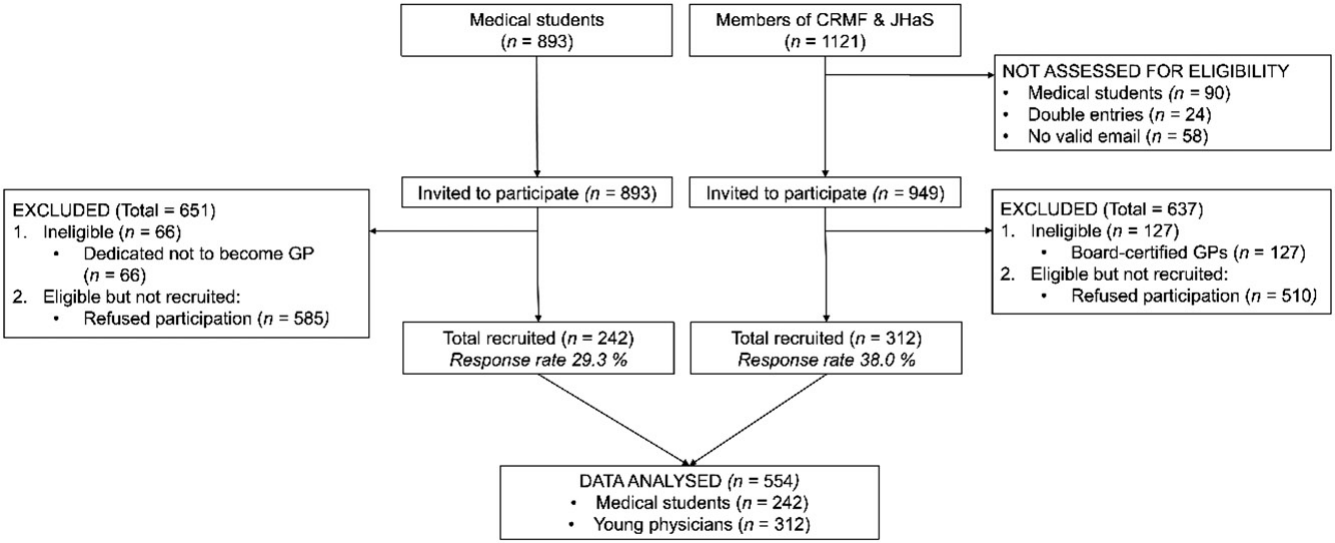
Flow chart.

To represent young physicians, we surveyed members of two societies, since there is no register of future GPs in Switzerland. Swiss Young General Practitioners Association (JHaS) members see themselves as future GPs. Most are in training, since the association recruits its members among students and residents. GPs can remain members for only five years after they begin practicing. We sent the survey to the 595 registered members, and excluded current medical students. Cursus Romand de Médecine de Famille (CRMF), a multiregional umbrella association of general practice training programs in the French-speaking part of Switzerland, pre-selected 400 of 526 current members by excluding all their medical student members. In the 24 cases of overlapping membership, we contacted potential participants only through JHaS. We later excluded all board-certified GPs (*n* = 127), since our goal was to target the population already or potentially eligible for training (Figure 1).

To recruit participants, JHaS and CRMF members received an email. Students were invited by email sent by the student coordinating center in Bern, Basel, and Lausanne. The invitation to the questionnaire was published in the medical student magazine in Zürich, and distributed on paper flyers and by email in Geneva (according to the regulations of each university). To make participation more attractive, we invited all participants to enter a lottery. Two reminders were sent to non-responders in the JHaS and CRMF groups. Universities limited us to one reminder for the student group.

The questionnaire was available in German and French, the languages spoken by 86% of Swiss residents [17]. It was hosted on SurveyMonkey® (www.surveymonkey.com, Palo Alto, CA, USA).

### The GO-GP curriculum

We drafted a model curriculum by analyzing existing GP training programs (Table 1) in Switzerland [18]. These programs are very heterogeneous, given the fact, that Switzerland is a federalist country. We then looked at evaluations of those programs, if available [5,19,20]. We extracted recommendations from the literature on postgraduate training [21–25]. We synthesized from these a model to meet the criteria of the Swiss Institute of Medical Education (SWIF) criteria for board certification [13].

**Table 1.** Postgraduate GP training programs in Switzerland.

Canton	Curriculum	PA	Requisite years in practice before admission (specialty)	Length of Curriculum (in years)	Post-degree commitment to practice	Availability of part-time work
AG	yes	yes	no	5	(yes)**	-*
BE	no	yes	3 (internal medicine/PA)	-*	no	(yes)
BL	(yes)***	yes	-*	–	-*	-*
BS	(yes)***	yes	-*	-*	-*	-*
FR	(yes)***	yes**	no	-*	-*	-*
GR	yes	yes	no	4-5	(yes)*	-*
GE	yes	yes**	2 (internal medicine)	1-3	no	yes
JU	yes	yes**	no	5	no	yes
LU	yes	yes	3 (min 2 internal medicine)	2	(yes)	-*
NE	yes	yes**	no	no	no	yes
SH	yes	yes	0-3	no	no	(yes)
SG	yes	yes	2-3	½ - 2	yes	yes
VD	yes	yes**	no	2-5	no	(yes)
VS	yes	yes**	no	(5)	no	yes
ZH	yes	yes	2-3 (min 2 internal medicine)	2	yes	no
ZG	yes	yes	no	3	no	-*

*no information available ** non-binding/binding declaration of intent, *** in development, PA = training module in general practice.

All cantons, except Ticino, offer training modules in GP practices. Postgraduate training programs in general practice are widely implemented.

We defined “curriculum” as an institutionalized process of teaching and learning that leads to a specialization. A curriculum may include internships [26], so we added training modules in practices to our model goal-oriented curriculum in general practice (GO-GP) [5,21,27]. GO-GP also included traditional medical teaching (e.g., lectures) and a peer-based learning community that met regularly, participants could see if they had met their learning goals [22,28]. Some of the learning goals in GO-GP would be determined by the Swiss Society of General Internal Medicine (SSGIM), some by the institute of primary care that implements the curriculum. GO-GP trainees can also choose some of their own learning goals [22,29]. Every young physician in GO-GP would also regularly discuss career goals with their mentor [30], who will keep them on track and help them solve problems. Career goals shape a trainee’s progress through GO-GP [29], so GO-GP is flexible enough to adapt to preferences, for example, for part-time work, to accommodate family planning [31,32].

### Processes and outcomes

We administered a three-part (A-C) cross-sectional, anonymous online survey on March 6^th^ 2017.

We designed three different versions of Part A, tailored to each survey population. All contained questions about personal characteristics, including gender and birth year. All asked the likelihood of becoming a GP (10-point Likert scale). The versions for JHaS and CRMF members asked participants their current occupation, workload, and canton of work [33]. The version for medical students asked which university they attended and their home canton. We asked students how likely it was they would become a GP (determined to become GP; GP positive; undecided; determined to become something other than GP) [4]. Students who either did or did not want to become a GP were asked what point in time they made that decision (undecided and GP positive students were not asked this question). Those determined not to become GPs were excluded. We excluded them, since our target population were students who were potentially available for postgraduate training in general practice.

Part B and C were the same in all surveys. Part B asked participants to rate their interest in a curriculum, if a curriculum would make becoming a GP more attractive, and if they thought it would encourage more medical students to become GPs. Participants also rated nine general features of a postgraduate curriculum, and seven requirements (5-point Likert scale). We asked them to choose the seven most important rotations from a list we provided. They also chose the three most important elective courses and certificate programs.

In Part C, we presented our GO-GP curriculum (Appendix 1). We asked participants to rate the whole curriculum on its attractiveness (10-point Likert scale). We asked participants how much the GO-GP program would motivate them to become a GP (5-point Likert scale). We also asked if they thought the GO-GP curriculum would help them advance faster, make them more self-confident in their practice, or make becoming GP more attractive to the younger generation, and if it would raise the quality of GP training. We ended with two open questions: Which were the best features of GO-GP? Which features need improvement?

### Statistical analysis

For categorical data, we used the Chi-squared test. For continuous data, we used either t-test or Wilcoxon-Ranksum test to describe baseline characteristics and to capture the participants’ beliefs about what is important in a curriculum. We grouped participants into young physicians and medical students.

We calculated means and 95% confidence intervals (CI) for the attractiveness of the GO-GP program and stratified for stage of education, gender, age, region, and likelihood of becoming GP. We used simple linear regression models for the primary exposure (attractiveness of GO-GP) in part B, and the outcome (motivation to become GP) in parts A and C. We used a causal modelling approach to see if variables from parts A and B were eligible to be confounders or effect modifiers on the outcome. Those eligible were included in a multivariable linear regression model.

We used qualitative techniques to analyze the open questions about GO-GP in part C. We coded and categorized answers to our open question.

(Which were the best features of GO-GP? Which features need improvement?). The first author coded the answers and built categories in consultation with the last author; rare disagreements were resolved by consensus.

A two-sided p-value of 0.05 was statistically significant. We analyzed all data in STATA release 15.0 (Stata Corp, College Station, TX, USA).

## Results

### Baseline characteristics

We sent the survey to 949 JHaS/CRMF members. Response rate among young physicians was 38% (*n* = 312), after excluding board-certified GPs. (Figure 1). The survey also went to 893 medical students; response rate was 29.3% (*n* = 242), after excluding students who did not want to become GPs.

French speakers made up 44%, and German speakers 56%. In the general population, 23% speak French and 63% speak German, so we included a disproportionately high number of French-speakers [17]. Our respondents came from all regions of Switzerland [33], and from every university that offers a Master’s in medicine. Women made up 66% of all participants, but only 59% of medical students. Mean age was 29.5 years. Most respondents were young physicians (56%) (Table 2). A large majority said they were very likely to become GPs (median: 8 of 10 points). Medical students and young physicians differed significantly (7 vs. 9 points; *p* < 0.001).

**Table 2.** Baseline characteristics.

Baseline characteristics (%, per column)	Overall n = 554	Undergraduates n = 242	Postgraduates n = 312	P-value^a^			
Likelihood of becoming GP, median (IQR)	8 (7-9)	7 (5-8)	9 (7-10)	<0.001			
Female, n (%)	352 (66)	128 (59)	224 (72)	0.002			
Age, mean (SD)	29.5 (4.75)	25.9 (2.42)	32 (4.35)				
Language, n (%)				0.009			
German	311 (56)	151 (62)	160 (51)				
French	243 (44)	91 (38)	152 (49)				
Region^b^, n (%)				<0.001			
Lake Geneva region	174 (33)	67 (31)	107 (34)				
Swiss plateau	153 (29)	46 (21)	107 (34)				
Northwestern Switzerland	58 (11)	30 (14)	28 (9)				
Zurich	69 (13)	37 (17)	32 (11)				
Eastern Switzerland	39 (7)	20 (9)	19 (6)				
Central Switzerland	29 (5)	11 (5)	18 (6)				
Ticino	8 (2)	7 (3)	1 (0)				
University^c^, n (%)							
Bern		55 (25)					
Basel		30 (14)					
Zurich		52 (24)					
Geneva		47 (21)					
Lausanne		34 (16)					

SD = standard deviation.

IQR = interquartile range.

a Chi 2 for categorical data, t-test for normally distributed continuous data, Wilcoxon Rank-Sum test for not-normally distributed data.

b work region for postgraduates, home region for undergraduates.

c asked in undergraduates only.

### Desirable features of a curriculum

**Figure 2.**
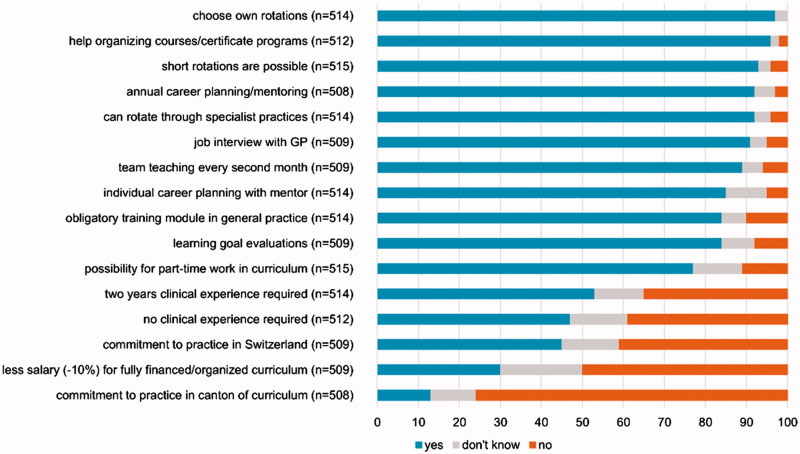
Desirable features for a curriculum in general practice training (sorted for yes, don’t know and no in percent).

Young physicians and medical students generally chose the same desirable and undesirable features in a post-graduate training program (Figure 2). The exception was that only 45% of medical students thought it necessary to have two years of clinical experience before entering a training program; but 58% of young physicians felt this much clinical experience was necessary. Over 90% of both groups rated these features as most desirable: choose own specialty rotations; guidance in choosing courses/certificate programs and administrative help; access to short rotations in specialties; meet with mentor for a yearly career planning session; rotations in specialist practices; and, job interview with a GP. 45% of respondents agreed that young physicians should commit to practice in Switzerland after completing their training program, and fewer (13%) thought it desirable to ask for a commitment to practice in the canton where they were trained. 30% agreed that they should contribute 10% of their salary for participating in a fully financed and organized postgraduate training program. Young physicians were significantly more likely to agree to the salary cut (34%) than medical students (23%).

**Figure 3.**
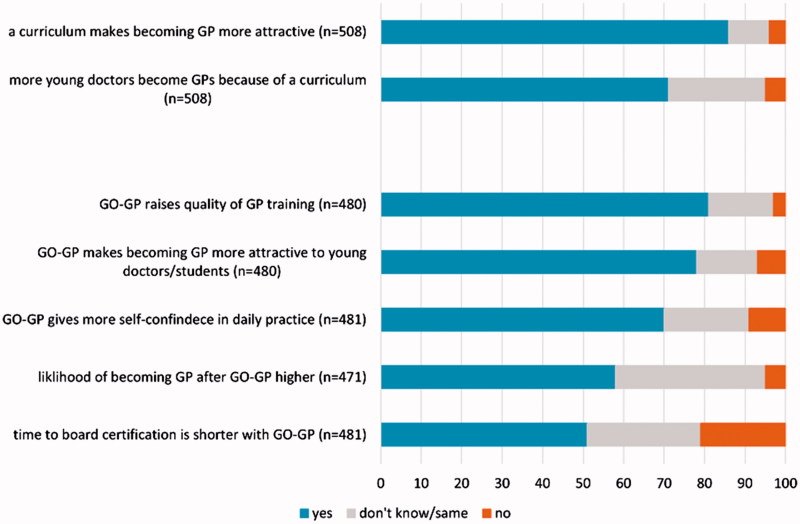
Opinions on effects of curricula in general and of GO-GP in particular (in percent).

Differences were statistically significant for five other features, but these differences were mostly a matter of degree. Agreement between young physicians was strong, medical students agreed, but not as strongly. Medical students were not as much in accord about the need for an obligatory GP training module, or about the need for an option for part-time work and training, individual career planning, a job interview with a GP, or evaluating learning goals.

When we stratified by region, respondents from the French-speaking part of Switzerland were more likely to reject the commitment within five years of completing the program to practice in the canton where they were trained (*p* = 0.022), or even in Switzerland (*p* < 0.001). The commitment to practice in Switzerland was rejected in the largest regions, where we had the most respondents (Lake Geneva region/Swiss plateau). In the smaller regions, over 50% would accept a commitment to practice in Switzerland (Appendix 2).

Respondents listed these seven specialties as the most important to include in a curriculum: dermatology/venereology; oto-rhino-laryngology; emergency medicine; psychiatry/psychotherapy; surgery; rheumatology; geriatrics (Appendix 3). The three most desired courses/certificate programs were ultrasound, laboratory medicine, and manual medicine (Appendix 4). Respondents generally agreed that a curriculum would make becoming a GP more attractive, and that it would increase the number of young physicians who become GPs (Figure 3).

**Figure 4.**
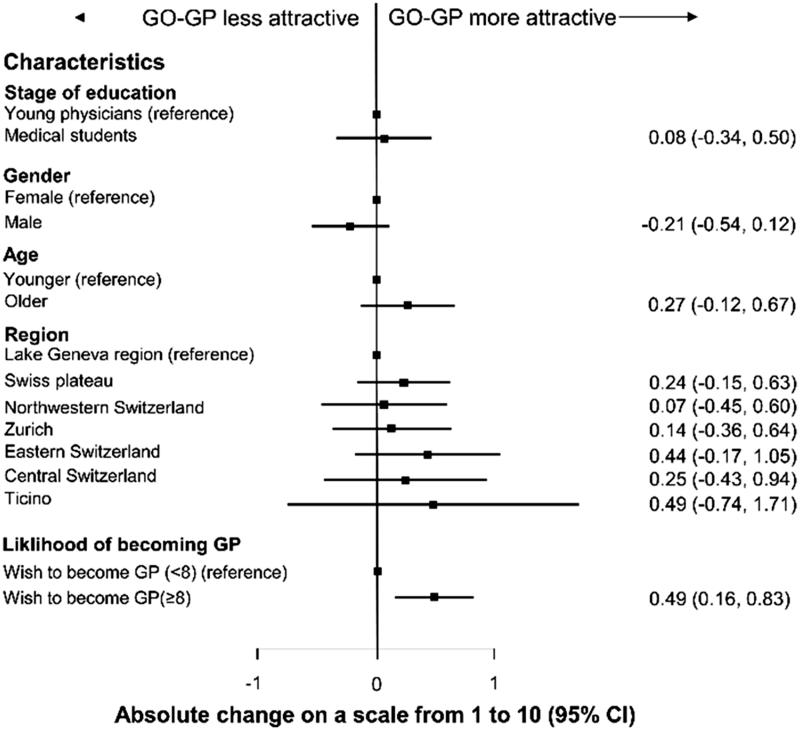
Attractiveness of GO-GP stratified for characteristics.

### Assessment of the model curriculum GO-GP

The overall attractiveness of our model GO-GP was rated 7.3/10, (95% IC 7.2–7.5). Participants who felt they were most likely to become GPs (those who rated the likelihood 8 or higher than the median of 8) found the curriculum significantly more attractive (Figure 4). After we stratified for other characteristics, we found no other statistically significant differences. Medical students and young physicians were similarly positive about the GO-GP Curriculum (Figure 4). Most (81%) believed GO-GP would improve GP training; 78% thought it would attract more people to the GP specialty; 70% thought it would make physicians more self-confident in future practice. Overall, 58% believed they would be more likely to become GPs if they could participate in a postgraduate training program like GO-GP. Half the participants believed GO-GP could shorten the time to board certification (Figure 3).

Access to short rotations was mentioned most frequently (*n* = 169) in the open questions where respondents could expand on the good features of GO-GP. The availability of training in the various specialties required for general practice (*n* = 103) came next, followed by a well-organized training framework (*n* = 89), and continuous mentoring (*n* = 82). These were the same features respondents generally rated positively in a postgraduate training program.

**Figure 5.**
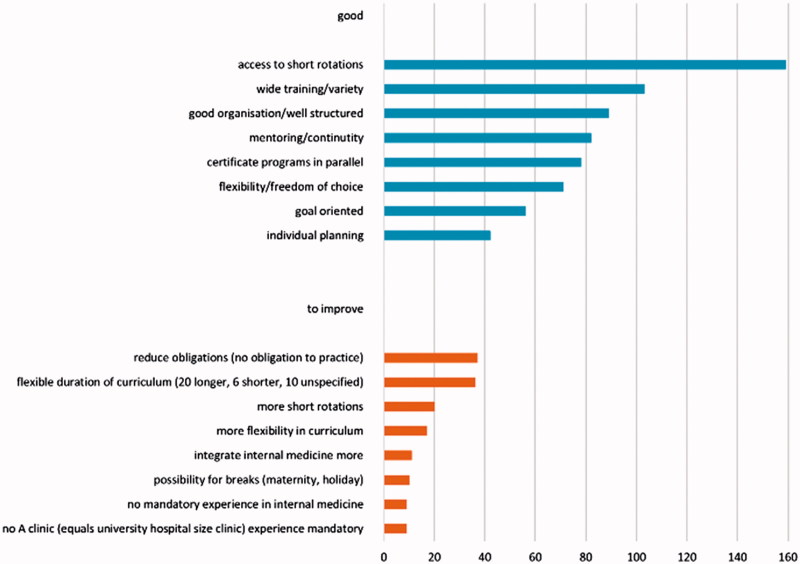
Good features and features to improve in GO-GP (absolute number of indications).

Respondents made a range of suggestions for improving GO-GP. The need to reduce obligations (*n* = 37) like commitment to practice, and the ability to adjust the length of the program (*n* = 36) (for example, finishing in two years instead of three), were the most frequently mentioned (Figure 5).

## Discussion

### Summary

The idea of becoming a GP was very attractive to our participants (8/10 on a Likert scale), when excluding students who were determined not to become GPs (*n* = 66). Young physicians generally agreed on which attractive features should be part of a postgraduate training program. Medical students did not agree as strongly, but this was a difference of degree, not direction. Young physicians may have answered more consistently because most had already chosen their specialty and had work experience, while medical students were less likely to have either. The features participants liked in a general curriculum were also attractive in our GO-GP model. Overall, participants were attracted to the GO-GP program. Those most strongly motivated to become GPs were significantly more attracted to GO-GP. Most respondents believed GO-GP would benefit young physicians, attract more young physicians to a career in general practice and improve the quality of their training.

### Strengths and limitations

Our sample of young physicians and medical students was drawn from all regions of Switzerland, and we successfully reached the population most interested in the GP specialty. Though respondents were heterogeneous, their responses were consistent, which suggests our questionnaire was easy to understand. We were limited by a low response rate as often seen in physician surveys (38% for young physicians and 27% for medical students). Because we could not compare responders to non-responders, we cannot eliminate the risk of selection bias. Because CRMF is a large program in mostly French-speaking Cantons, French speakers were overrepresented (23% in the general population; 44% in our sample) [17]. Most Swiss studies underrepresent French speakers, so we were glad to capture their opinions. Our results suggest that the disproportion did not increase bias because French and German speakers gave similar answers in most cases. There was a higher percentage of women (59% of medical students; 72% of young physicians) in our sample when compared to general percentages of women at universities (51.8%) [34]. In the medical field though in 2016, women earned 62% of specialist titles for general internal medicine and made up 56% of medical school graduates in Switzerland [32]. The trend towards more women in the profession is clear, thus, we believe our sample represents this future trend and is therefore not a limitation.

### Literature and discussion

In general, countries that do not require patients to first contact their primary care physicians, for example Germany and Austria [6], find it more difficult to recruit prospective GPs and to retain them. In contrast, in countries like the Netherlands, where training is structured and healthcare is rooted in general practice, the shortage is less severe. By introducing organized training programs, countries like Germany, Austria and Switzerland can encourage young physicians and support them towards becoming GPs, by giving a clear role and image to the specialty of general practice [8,25].

The desire of young GPs to be taught in GP practice and other outpatient settings meets our observation that in Switzerland GP training programs have increasingly moved out of hospitals and into practices, so more and more training is taking place in the practice environment. Given the different epidemiology of patients in practice this is very important.

Most respondents thought regular mentoring should be integrated into the curriculum, which aligns with findings from other studies and evaluations [5,23,30,35]. An unpublished survey of young physicians found over 75% wanted to participate in a mentoring program; two-thirds of young physicians would even pay for such a program [35].

Respondents from all regions rejected the commitment to practice in the canton where the curriculum was offered. Few participants were willing to commit to settling in the canton within five years of completing the program. In French-speaking regions, more respondents rejected the commitment to practice. Some existing curricula in Switzerland still include several binding elements; other curricula have dropped them. The most common is an agreement to commit to practice in the canton that offers the curriculum [18]. Young physicians from cantons where doctors had experience with binding requirements might be prejudiced against such commitments. Similarly, a less restrictive commitment to practice anywhere in Switzerland was still rejected. We explored differences of opinion between participants from the various regions, and as before, the French-speaking regions were most likely to reject the commitment. However, many participants were also undecided. Therefore, we cannot make a strong claim that most respondents rejected this binding element.

We were also curious if young physicians were willing to help finance their own training curriculum by taking a 10% salary cut to subsidize the institutions that trained them. Half our respondents rejected that proposal, 30% accepted it, and 20% were undecided.

### Future

We intend to pilot an improved version of our GO-GP curriculum in a real-world setting. Respondents made some suggestions for improving GO-GP such as for a more flexible curriculum that takes the accumulated work experience of participants into account. We initially decided trainees would be eligible for GO-GP after two years of mandatory work experience. This now seems too strict, and in some cases, infeasible. We will continue to require at least a year of work experience in general internal medicine, but individually adjust specialty rotations (with a focus on outpatient work). To improve GO-GP, we will work with clinics to create contracts that support these goals, and make sure they can meet the learning goals of GP trainees.

When we implement the pilot program, we will simultaneously aim to conduct a longitudinal study among the new student population to see the program’s effects, along with a careful evaluation program for participants. Despite clear lack of enthusiasm for binding elements, we do not think imposing binding elements will drive interested young physicians away from GO-GP, as long as the commitments are not too strict. A certain degree of commitment can help to persuade policy makers to financially support GO-GP, while also take young physicians preferences and interests into account. Most segments of the GO-GP curriculum already are being offered by Swiss institutions, and pose no unusual financial burden. Existing resources can be integrated into a curriculum, but we will have to identify a funding mechanism finance the focused framework and continuous mentoring. Our findings go in line with a systematic review on training standards statements of family medicine postgraduate training [23].

## Conclusion

Overall, medical students and young physicians found similar features attractive in the general and GO-GP curriculum, regardless of region or gender, and thought an attractive curriculum would attract more young doctors to the GP specialty.

## Declarations

### Availability of data and materials

The datasets used and analyzed during the current study are available from the corresponding author on reasonable request.

## Appendix Appendix 2. Acceptance of commitment to practice

**Table:**

Region (%, per column)^a^	Commitment to practice in CH Indicating yes	Commitment to practice in Canton of curriculum Indicating yes	p-value
Geneva lake region (n = 163)	54 (23)	13 (8)	
Swiss plateau (n = 149)	61 (41)	30 (20)	
Northwestern Switzerland (n = 57)	32 (56)	8 (14)	
Zurich (n = 66)	36 (55)	5 (8)	
Eastern Switzerland (n = 37)	23 (61)	5 (13)	
Central Switzerland (n = 28)	20 (71)	6 (21)	
Ticino (n = 8)	5 (62)	0 (0)	
French part vs. other parts			
commitment to practice in CH			<0.001
commitment to practice in Canton of curriculum			0.02

a number/percentage indicating yes.
